# Multi-Layered Porous Helmholtz Resonators for Low-Frequency and Broadband Sound Absorption

**DOI:** 10.3390/ma19030600

**Published:** 2026-02-04

**Authors:** Xuewei Liu, Tianyu Gu, Ling Li, Dan Wang

**Affiliations:** 1 School of Mechanical and Electrical Engineering, Xi’an University of Architecture and Technology, Xi’an 710055, China; 15129603033@163.com; 2 Xi’an Key Laboratory of Intelligent Technology for Heavy Machinery Equipment, Xi’an University of Architecture and Technology, Xi’an 710055, China; 3 Xi’an Thermal Power Research Institute Co., Ltd., Xi’an 710054, China; wd8625701371@163.com

**Keywords:** sound absorption, porous Helmholtz resonator, double porosity material, Helmholtz resonance

## Abstract

Unlike classical multi-layered micro-perforated panels (MPPs), which rely on sub-millimeter orifices for sound dissipation, we propose a multi-layered porous Helmholtz resonators absorber. It consists of alternately layered perforated porous material panels and perforated rigid panels with millimeter- to centimeter-scale orifices, primarily relying on porous materials for sound energy dissipation. Theoretically, perforated porous material panels are modeled as homogeneous fluid layers using double porosity theory, and the total surface impedance is derived through bottom-to-top impedance translation. A double-layered prototype was tested to validate the theoretical and numerical models, achieving near-perfect absorption peaks at 262 Hz and 774 Hz, with a subwavelength total thickness of 11 cm and a broadband absorption above an absorption coefficient of 0.7 from 202 Hz to 1076 Hz. Simulations of sound pressure, particle velocity, power dissipation, and sound intensity flow confirm that Helmholtz resonances in each layer enhance sound entry into resistive porous materials, causing absorption peaks. Parameter studies show this absorber maintains high absorption peaks across wide ranges of orifice diameters and panel thicknesses. Finally, an optimized triple-layer porous Helmholtz resonators absorber achieves an ultra-broadband absorption above a coefficient of 0.95 from 280 Hz to 1349 Hz with only 16.5 mm thickness. Compared with conventional MPPs, this design features significantly larger orifices that are easier to fabricate and less susceptible to blockage in harsh environments, offering an alternative solution for low-frequency and broadband sound absorption.

## 1. Introduction

Sound-absorbing materials are employed to mitigate noise, serving to protect human health [1] and prevent acoustic damage to precision instruments [2]. Micro-perforated panels (MPPs) [3,4] are among the most widely used absorbing materials owing to their good low-frequency absorption performance. They are usually fabricated by perforating orifices in a panel and then adding an air cavity between the perforated panel and the rigid backing. When the incident sound wavelength is much larger than the thickness of the back air cavity, the MPP can be seen as parallel Helmholtz resonators [5] (i.e., mass-spring vibration systems): the air in the small orifice and in the back cavity act roles as air masses and air springs, respectively. Maa [3,4] first validated that these Helmholtz resonators can transform into perfect sound absorbers when the orifice diameter decreases to the sub-millimeter scale. At the Helmholtz resonance frequency, significant energy dissipation occurs around the small orifices owing to the dominant viscous effects and secondary thermal effects, as the incident sound wave propagates through the orifices. Based on this mechanism, many sound-absorbing metamaterials have been proposed to lower the absorption peak frequency (i.e., Helmholtz resonance frequency), mainly by extending the orifice into the back air cavity to increase the air mass [6,7,8] or coiling up the back air cavity [9,10,11] to decrease the air spring stiffness.

To broaden the absorption bandwidth, parallel connection and series connection of different Helmholtz resonators are adopted to form a multiple-resonance system, leading to multiple absorption peaks. Wang et al. [12] realized multiple perfect sound absorption peaks through the parallel connection of multiple non-perfect Helmholtz resonance absorbers. Peng et al. [13] proposed a detailed method from the perspective of surface impedance to design parallel Helmholtz resonators with multiple nearly perfect absorption peaks. The parallel connection of Helmholtz resonators with extended necks [14] or coiled-up back air cavities [15] can further broaden the low-frequency sound absorption bandwidth.

Compared with aforementioned Helmholtz resonators in parallel, Helmholtz resonators in series are more straightforward to fabricate, as there is no need to add partitions between the resonators. They can be readily implemented using multi-layer micro-perforated panels [16,17], albeit at the cost of increased vertical thickness. The effective absorption bandwidth at low frequencies was usually further broadened by oblique necks [18,19] and extended necks [20]. Helmholtz resonators in both parallel and series can further improve the bandwidth of sound absorption [21,22,23]. In demand of mechanical strength, researchers perforated orifices in the walls of honeycomb–corrugation hybrid cores [24] and face-centered cubic cores [25] to realize the parallel assembly and cascade of different Helmholtz resonators simultaneously to broaden the effective absorption band. Recently, the combination of multi-layered micro-perforated panels and sonic black holes was studied to further improve the sound absorption performance [26,27].

Different from above, Helmholtz resonators dissipate incident sound energy by thermoviscous effects around the small orifices. Liu et al. [28] proposed a porous Helmholtz resonator composed of a perforated panel with orifices of centimeter scale backed with perforated porous materials. The air in an orifice of the perforated panel acts as an “air mass”, and the air in a perforation hole of the perforated porous material acts as an “air spring”. At the Helmholtz resonance frequency, most of the incident sound energy intensely diffuses into the resistive perforated porous materials, leading to strong dissipation and perfect sound absorption. In comparison with the perforated porous materials [29,30], the addition of a perforated panel can substantially enhance the low-frequency sound absorption. In comparison with the conventional Helmholtz resonators with orifice diameters of sub-millimeter scale, the larger orifices of porous Helmholtz resonators are easier to fabricate and less susceptible to blockage in harsh environments. The parallel connection of these porous Helmholtz resonators was designed to broaden the effective absorption bandwidth [31,32].

Compared with the parallel connection of porous Helmholtz resonators [31,32], the series connection in a multi-layer form proposed in this paper is more straightforward to fabricate, as there is no need to add partitions between the resonators. Moreover, when the planar installation space is dimensionally constrained, a multi-layer configuration is more applicable than a parallel arrangement. Therefore, the absorption performances and mechanisms of multi-layered porous Helmholtz resonators are investigated in this study. A theoretical model mainly based on the double porosity theory [33] and the bottom-to-top impedance translation method for this multi-layer absorber is proposed in Section 2 and then is validated by numerical simulations in Section 3 and experimental measurements in Section 4 using a double-layer test sample. Sound absorption performances and mechanisms are discussed in Section 5. Parameter studies are conducted in Section 6, and an optimized triple-layered porous Helmholtz resonators absorber is presented in Section 7.

## 2. Theoretical Model Developing

**Figure 1:**
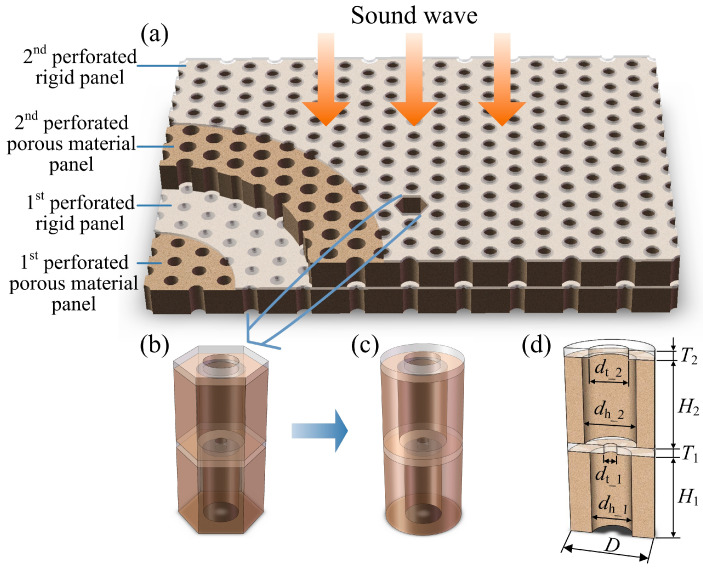
(**a**) Double-layered porous Helmholtz resonators absorber; (**b**) an extracted representative hexagonal cell; (**c**) an equivalent cylindrical cell having equal volume with the hexagonal cell; (**d**) a semi-cell with dimensions.

The double-layered porous Helmholtz resonators absorber subjected to normal sound wave incidence is shown in Figure 1a, which is constructed by alternately layering different perforated porous material panels and perforated rigid panels. A representative hexagonal unit cell is extracted as shown in Figure 1b and is equivalent to a cylindrical cell as shown in Figure 1c, with equal volume for simplifying the finite element simulation from a three-dimensional model to a two-dimensional axisymmetric model. The dimensions are given in Figure 1d through a semi-cell with outer diameter D: from bottom to top, the perforated porous material panels have thicknesses $H_{1}$ and $H_{2}$, perforation hole diameters $d_{h\_1}$ and $d_{h\_2}$; the perforated rigid panels have thicknesses $T_{1}$ and $T_{2}$, perforation hole diameters $d_{t\_1}$ and $d_{t\_2}$. The acoustic properties of the porous material matrix are described by the Johnson–Champoux–Allard (JCA) model [34,35] with five parameters: porosity $\phi_{m}$, static airflow resistivity σ, tortuosity $\alpha_{\infty }$, viscous characteristic length Λ, and thermal characteristic length $\Lambda^{\prime }$.

Considering that the particle vibration velocity in the perforation holes of the thin perforated rigid panel is nearly uniform in the sound incidence direction [3,4], according to the impedance translation theorem, the surface impedance of the multi-layered Helmholtz resonator absorber is

(1) $$Z_{s\_i}=Z_{\mathrm{dp}\_i}\frac{Z_{\mathrm{dp}\_i}-jZ_{s\_i-1}\cot\left(k_{\mathrm{dp}\_i}H_{i}\right)}{Z_{s\_i-1}-jZ_{\mathrm{dp}\_i}\cot\left(k_{\mathrm{dp}\_i}H_{i}\right)}+Z_{\mathrm{prp}\_i},\;$$

where $Z_{s\_i}$ is the surface impedance at the upper surface of the ith perforated rigid panel; $H_{i}$, $Z_{\mathrm{dp}\_i}$, and $k_{\mathrm{dp}\_i}$ are the thickness, equivalent characteristic impedance, and wavenumber of the ith perforated porous material panel, respectively; j is the imaginary unit; and $Z_{\mathrm{prp}\_i}$ is the impedance of the ith perforated rigid panel expressed as [36]:

(2) $$Z_{\mathrm{prp}\_i}=\frac{j\omega \rho_{0}T_{i}}{\phi_{\mathrm{prp}\_i}}\left[1-\frac{2J_{1}\left(y\sqrt{-j}\right)}{y\sqrt{-j}J_{0}\left(y\sqrt{-j}\right)}\right]+2\frac{\sqrt{2\omega \rho_{0}\eta }}{\phi_{\mathrm{prp}\_i}}+2\frac{j\omega \rho_{0}\varepsilon_{i}}{\phi_{\mathrm{prp}\_i}},\;$$

where $y=d_{t\_i}\sqrt{\rho_{0}\omega /\eta }/2$, ω is the angular frequency; $\rho_{0}=1.2$ kg/m^3^ and $\eta =1.84\times 10^{-5}$ Pa·s are the density and dynamic viscosity of air, respectively; $T_{i}$, $d_{t\_i}$, and $\phi_{t\_i}=d_{t\_i}^{2}/D^{2}$ are the thickness, perforation diameter, and perforation ratio of the ith perforated rigid panel, respectively; $J_{0}$ and $J_{1}$ are the first kind Bessel functions of zero and first order, respectively; $2\sqrt{2\omega \rho_{0}\eta }/\phi_{t\_i}$ [5] is the additional acoustic resistance owing to the friction on the upper and lower surfaces of the perforated rigid panel around the perforation hole; and $2j\omega \rho_{0}\varepsilon_{i}/\phi_{t\_i}$ is the additional reactance considering the wave distortion when sound enters and leaves the perforation hole, with the end correction length $\varepsilon_{i}$ for the ith perforated rigid panel expressed as [37]:

(3) $$\varepsilon_{i}=0.425d_{t\_i}\left(1-1.41\sqrt{\phi_{t\_i}}+0.34{\sqrt{\phi_{t\_i}}}^{3}+0.07{\sqrt{\phi_{t\_i}}}^{5}\right).$$

In Equation (1), the equivalent characteristic impedance $Z_{\mathrm{dp}\_i}$ and wavenumber $k_{\mathrm{dp}\_i}$ of the ith perforated porous material panel are calculated by

(4) $$Z_{\mathrm{dp}\_i}=\sqrt{\rho_{\mathrm{dp}\_i}K_{\mathrm{dp}\_i}},$$

(5) $$k_{\mathrm{dp}\_i}=\omega \sqrt{\rho_{\mathrm{dp}\_i}/K_{\mathrm{dp}\_i}},$$

where $\rho_{\mathrm{dp}\_i}$ and $K_{\mathrm{dp}\_i}$ are the equivalent density and bulk modulus of the ith perforated porous material panel, respectively, calculated using the double porosity theory [33] regarding the panel as a homogenous fluid medium:

(6) $$\rho_{\mathrm{dp}\_i}={\left[\rho_{m}^{-1}+\left(1-\phi_{h\_i}\right)\rho_{p\_i}^{-1}\right]}^{-1},$$

(7) $$K_{\mathrm{dp}\_i}={\left[K_{m}^{-1}+\left(1-\phi_{h\_i}\right)W_{i}\cdot K_{p\_i}^{-1}\right]}^{-1},$$

where $\phi_{h\_i}=d_{h\_i}^{2}/D^{2}$ is the perforation ratio of the ith perforated porous material panel; $\rho_{m}$ and $K_{m}$ are the effective density and effective bulk modulus of the porous material matrix, respectively; and $\rho_{p\_i}$ and $K_{p\_i}$ are the effective density and effective bulk modulus of a perforation hole structure (i.e., the porous material domain of the ith perforated porous material panel is replaced by rigid medium), respectively.

For the porous material matrix, its acoustic properties are described by the JCA model as in [34,35]:

(8) $$\rho_{m}=\frac{\alpha_{\infty }\rho_{0}}{\phi_{m}}\left(1+\frac{\phi_{m}\sigma }{j\omega \alpha_{\infty }\rho_{0}}\sqrt{1+j\frac{4\omega \eta \rho_{0}\alpha_{\infty }^{2}}{\phi_{m}^{2}\sigma^{2}\Lambda^{2}}}\right),$$

(9) $$K_{m}=\frac{\gamma P_{0}}{\phi_{m}}{\left[\gamma -\left(\gamma -1\right){\left(1+\frac{8\kappa }{j\omega {\Lambda^{\prime }}^{2}C_{p}\rho_{0}}\sqrt{1+\frac{j\omega {\Lambda^{\prime }}^{2}C_{p}\rho_{0}}{16\kappa }}\right)}^{-1}\right]}^{-1},$$

where γ=1.4, $P_{0}=101325$ Pa, κ=0.026 W/(m·K), and $C_{p}=1000$ J/(kg·K) are the ratio of specific heat, atmospheric pressure, thermal conductivity and specific heat capacity of air, respectively.

For the perforation hole structure, $\rho_{p\_i}$ and $K_{p\_i}$ are as follows [38]:

(10) $$\rho_{p\_i}=\frac{\rho_{0}}{\phi_{h\_i}}{\left[1-\frac{2}{s\sqrt{-j}}\frac{J_{1}\left(s\sqrt{-j}\right)}{J_{0}\left(s\sqrt{-j}\right)}\right]}^{-1},$$

(11) $$K_{p\_i}=\frac{\gamma P_{0}}{\phi_{h\_i}}{\left[1+\left(\gamma -1\right)\frac{2}{s\sqrt{-j}P_{r}}\frac{J_{1}\left(s\sqrt{-jP_{r}}\right)}{J_{0}\left(s\sqrt{-jP_{r}}\right)}\right]}^{-1},$$

where $s=d_{h\_i}\sqrt{\rho_{0}\omega /\eta }/2$, $P_{r}=\eta C_{p}/\kappa$ is Prandtl number.

$W_{i}$ in Equation (7) denotes the ratio of the average sound pressure in the porous material matrix of micro-pores to the average sound pressure in the macro-perforation hole of the ith perforated porous material panel. When the ratio of the macro-perforation hole size to the average micro-pore size is much smaller than 100, $W_{i}$ equals 1, which corresponds to the low permeability contrast case; when this ratio is much larger than 100, $W_{i}$ is a frequency dependent function corresponding to the high permeability contrast case, expressed as in [33]:

(12) $$W_{i}=1-\frac{j\omega P_{0}}{\phi_{m}K_{m}\omega_{d\_i}}{\left(\frac{j\omega P_{0}}{\phi_{m}K_{m}\omega_{d\_i}}+\sqrt{1+\frac{j\omega P_{0}}{\phi_{m}K_{m}\omega_{d\_i}}\cdot \frac{M_{d\_i}}{2}}\right)}^{-1},$$

where $\omega_{d\_i}=\left(1-\phi_{h\_i}\right)P_{0}/\left(E_{i}\sigma \phi_{m}\right)$ denotes the characteristic frequency of the sound diffusion effect, around which the sound pressure substantially diffuses from macro-perforation holes of the ith perforated porous material panel to the porous material matrix of micropores. $E_{i}=D^{2}\left[\ln\left(1/\phi_{h\_i}\right)-3/2+2\phi_{h\_i}-\phi_{h\_i}^{2}/2\right]/16$ [29,39] is an intrinsic geometrical parameter related to the static thermal permeability of an inverse structure of the ith perforated porous material panel (the porous material domain is replaced by air and macro-perforation holes are replaced by a rigid medium). $M_{d\_i}=8E_{i}/\left[\Lambda_{d\_i}^{2}\left(1-\phi_{h\_i}\right)\right]$ is a shape factor, $\Lambda_{d\_i}={2V_{i}/\Gamma }_{i}$, $V_{i}=\pi \left(D^{2}-d_{h\_i}^{2}\right)H_{i}/4$ is the volume of the porous material in a cylindrical cell of the ith perforated porous material panel, and $\Gamma_{i}=\pi d_{h\_i}H_{i}$ is the interface area between the porous material domain and air domain.

For the lower Helmholtz resonator, the surface impedance $Z_{s\_1}$ at the upper surface of the first perforated rigid panel is

(13) $$Z_{s\_1}=-jZ_{\mathrm{dp}\_1}\cot\left(k_{\mathrm{dp}\_1}H_{1}\right)+Z_{\mathrm{prp}\_1},$$

Then, $Z_{s\_2}$ for a double-layered Helmholtz resonator absorber can be obtained by Equation (1), and the absorption coefficient is expressed as

(14) α=4ReZs_2/z01+ReZs_2/z02+ImZs_2/z02,

where $z_{0}=\rho_{0}c_{0}$ is the characteristic impedance of air, $c_{0}=343$ m/s is the sound speed in air, and Re⋅ and Im⋅ denote the real part and imaginary part of a complex number, respectively. The surface impedance and sound absorption coefficient of an absorber with more layers can be calculated using the recurrence relation as shown in Equation (1).

## 3. Numerical Model Developing

**Figure 2:**
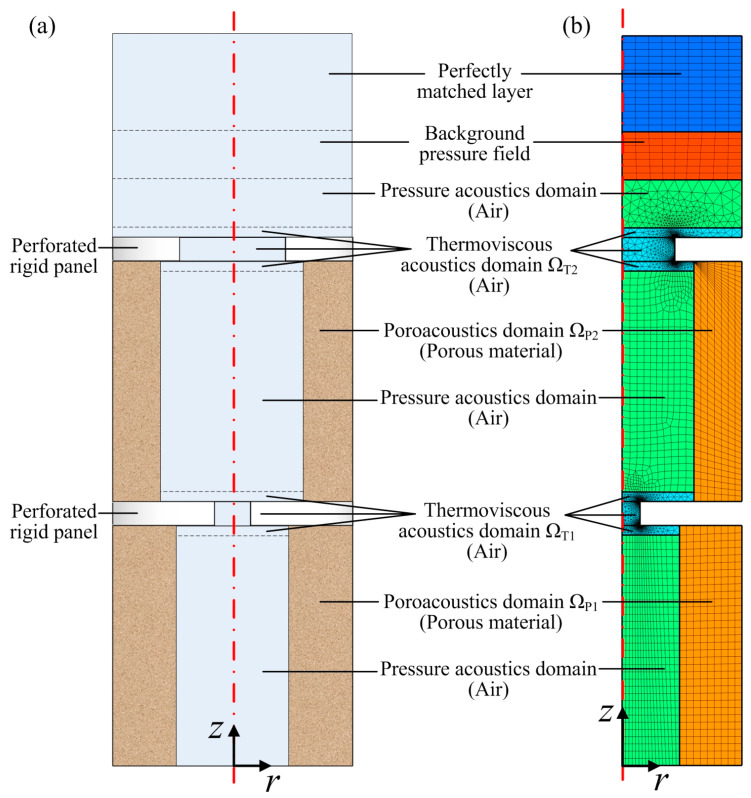
(**a**) Cross-section of the double-layered Helmholtz resonator; (**b**) two-dimensional axisymmetric finite element model.

To validate the developed theoretical model, a finite element model is constructed as shown in Figure 2 in the commercial software COMSOL Multiphysics 5.3. In this model, “Perfectly matched layer” acts as an anechoic end. “Background pressure field” supplies a downward incident plane sound wave with pressure amplitude of 1 Pa. “Pressure acoustics domain” is modeled as an air area where sound propagates without energy loss. “Poroacoustics domain” acts as air-saturated porous materials described by the JCA model, which are modeled in the pressure acoustics module of the software; $\Omega_{P1}$ and $\Omega_{P2}$ represent the areas of the porous material in the first layer and second layer, respectively. The air areas ($\Omega_{T1}$ and $\Omega_{T2}$) close to the rigid panel surfaces are modeled by “Thermoviscous acoustics domain” in the thermoviscous acoustics module of the software to consider the viscous and thermal energy dissipation when sound propagates in the two orifices of the two perforated rigid panels. It should be noted that, to improve the accuracy of the finite element simulation, four air layers above and below the perforated rigid panels are modeled by “Thermoviscous acoustics domain” to consider the energy dissipation owing to the friction on the panel surfaces, which corresponds to the added resistances shown in Equation (2).

The governing equation of the “Pressure acoustics domain” not considering the viscous and thermal energy dissipations in the software is

(15) $$\nabla^{2}p+\frac{\omega^{2}}{c^{2}}p=0$$

where p is the sound pressure.

The governing equation of the “Poroacoustics domain” in the software is

(16) $$\nabla^{2}p+\frac{\omega^{2}}{c_{m}^{2}}p=0$$

where $c_{m}=\sqrt{K_{m}/\rho_{m}}$ is the effective sound speed in the porous material.

The total sound power dissipation density (unit: W/m^3^) in the “Poroacoustics domain” is

(17) $$D_{P}=D_{P\_v}+D_{P\_t}$$

where $D_{P\_v}$ and $D_{P\_t}$ are the viscous power dissipation density and thermal power dissipation density, respectively, written as follows [40]:

(18) DP_v=12Im∇p⋅∇p*ωρm

(19) DP_t=−12Imωpp*Km

where the asterisk denotes the conjugate complex number.

The total sound power dissipation density (unit: W/m^3^) in the “Thermoviscous acoustics domain” is

(20) $$D_{T}=D_{T\_v}+D_{T\_t}$$

where $D_{T\_v}$ and $D_{T\_t}$ are the viscous power dissipation density and thermal power dissipation density, respectively. Appendix A shows the built-in governing equations of the “Thermoviscous acoustics domain” considering the viscous and thermal dissipations and the expressions of $D_{T\_v}$ and $D_{T\_t}$ in the software.

Therefore, the sound power dissipations in the four domains $\Omega_{P1}$, $\Omega_{P2}$, $\Omega_{T1}$, and $\Omega_{T2}$ are calculated by integrating the power dissipation density over these four domains, respectively:

(21) $$P_{P1}=\int D_{P}d\Omega_{P1}$$

(22) $$P_{P2}=\int D_{P}d\Omega_{P2}$$

(23) $$P_{T1}=\int D_{T}d\Omega_{T1}$$

(24) $$P_{T2}=\int D_{T}d\Omega_{T2}$$

The incident sound power is

(25) $$P_{\mathrm{in}}=\frac{S_{\mathrm{in}}p_{\mathrm{in}}^{2}}{2\rho_{0}c_{0}}$$

where $S_{\mathrm{in}}=\pi D^{2}/4$ is the area of the cell cross section perpendicular to the incident sound wave direction, and $p_{\mathrm{in}}$ is the sound pressure amplitude of the incident wave. Therefore, the energy dissipation ratios of the above four domains are

(26) $$\gamma_{P1}=P_{P1}/P_{\mathrm{in}}$$

(27) $$\gamma_{P2}=P_{P2}/P_{\mathrm{in}}$$

(28) $$\gamma_{T1}=P_{T1}/P_{\mathrm{in}}$$

(29) $$\gamma_{T2}=P_{T2}/P_{\mathrm{in}}$$

In the finite element model, the sound absorption coefficient can be obtained by first collecting the average sound pressure $p_{\mathrm{ave}}$ and average normal particle vibration velocity $u_{\mathrm{ave}}$ on the top surface of the entire structure, then calculating the surface impedance $Z_{s}=p_{\mathrm{ave}}/u_{\mathrm{ave}}$, and finally calculating the absorption coefficient α using Equation (14). Alternatively, it can also be calculated using energy dissipation:

(30) $$\alpha =\gamma_{P1}+\gamma_{P2}+\gamma_{T1}+\gamma_{T2}$$

## 4. Experiment Validation

**Figure 3:**
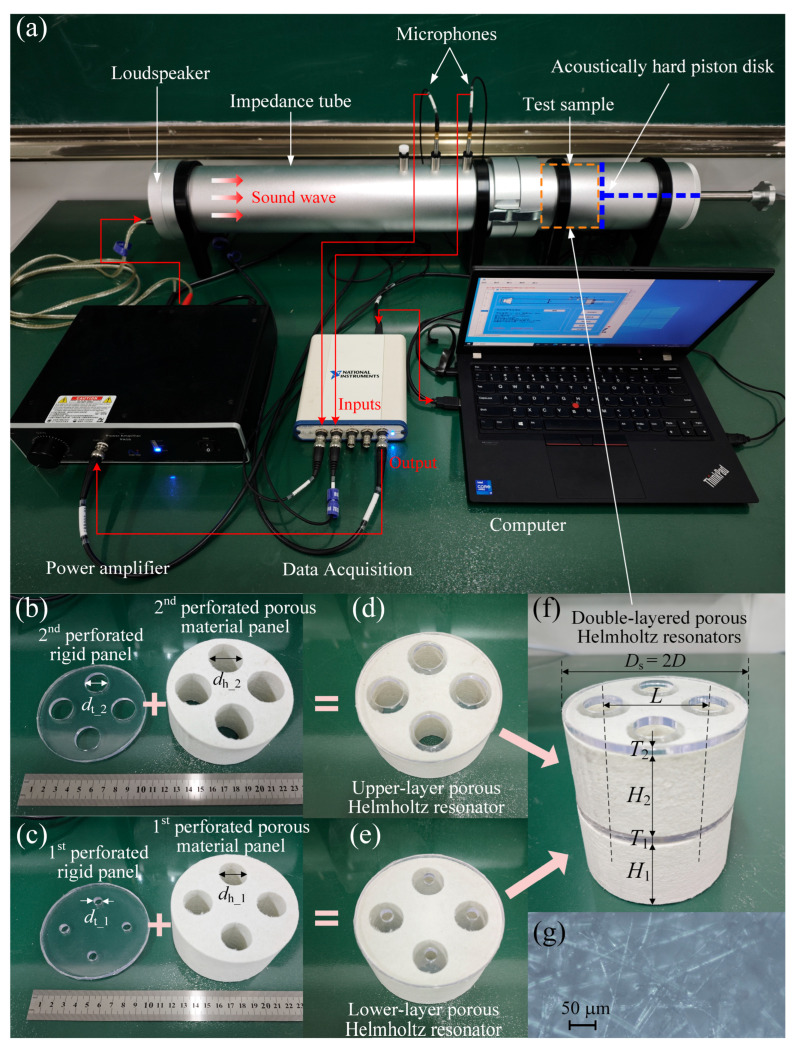
Sound absorption coefficient measurements: (**a**) experimental devices; (**b**–**f**): assembly process of the double-layered porous Helmholtz resonators; (**g**) optical micrograph of the porous material matrix made of ceramic fibers.

Sound absorption measurements are conducted in an impedance tube as shown in Figure 3a according to the standard “ASTM E1050-98” [41]. The assembly process of the double-layered porous Helmholtz resonators is shown in Figure 3b–f. The outer diameter of the test sample in Figure 3f is $D_{s}=2D=100$ mm to match the inner diameter of the impedance tube. Each layer of the sample has four evenly distributed perforation holes with a diagonal spacing of L=58 mm. Therefore, the sample can be regarded as a combination of four identical fan-shaped unit cells, each of which can be equivalent to a cylindrical unit cell with an outer diameter of D=50 mm. From the bottom to top of the sample, the perforated porous material panels have thicknesses $H_{1}=H_{2}=50$ mm and perforation hole diameters $d_{h\_1}=24$ mm and $d_{h\_2}=30$ mm; the perforated transparent rigid panels have thicknesses $T_{1}=T_{2}=5$ mm and perforation hole diameters $d_{t\_1}=7.5$ mm and $d_{t\_2}=22$ mm. The five JCA parameters of the porous material matrix made of ceramic fibers as shown in Figure 3g are porosity $\phi_{m}=0.89$, static airflow resistivity σ=305,767.3 N·s/m^4^, tortuosity $\alpha_{\infty }=3.7$, viscous characteristic length Λ=21.6 μm, and thermal characteristic length $\Lambda^{\prime }=53.6$ μm. Owing to the large ratio of the perforation hole diameters $d_{h\_1}=24$ mm and $d_{h\_2}=30$ mm to the micropore size (of the porous material matrix) which can be estimated by the thermal characteristic length, the perforated porous materials belong to the high permeability contrast case.

## 5. Sound Absorption Performances and Mechanisms Analysis

**Figure 4:**
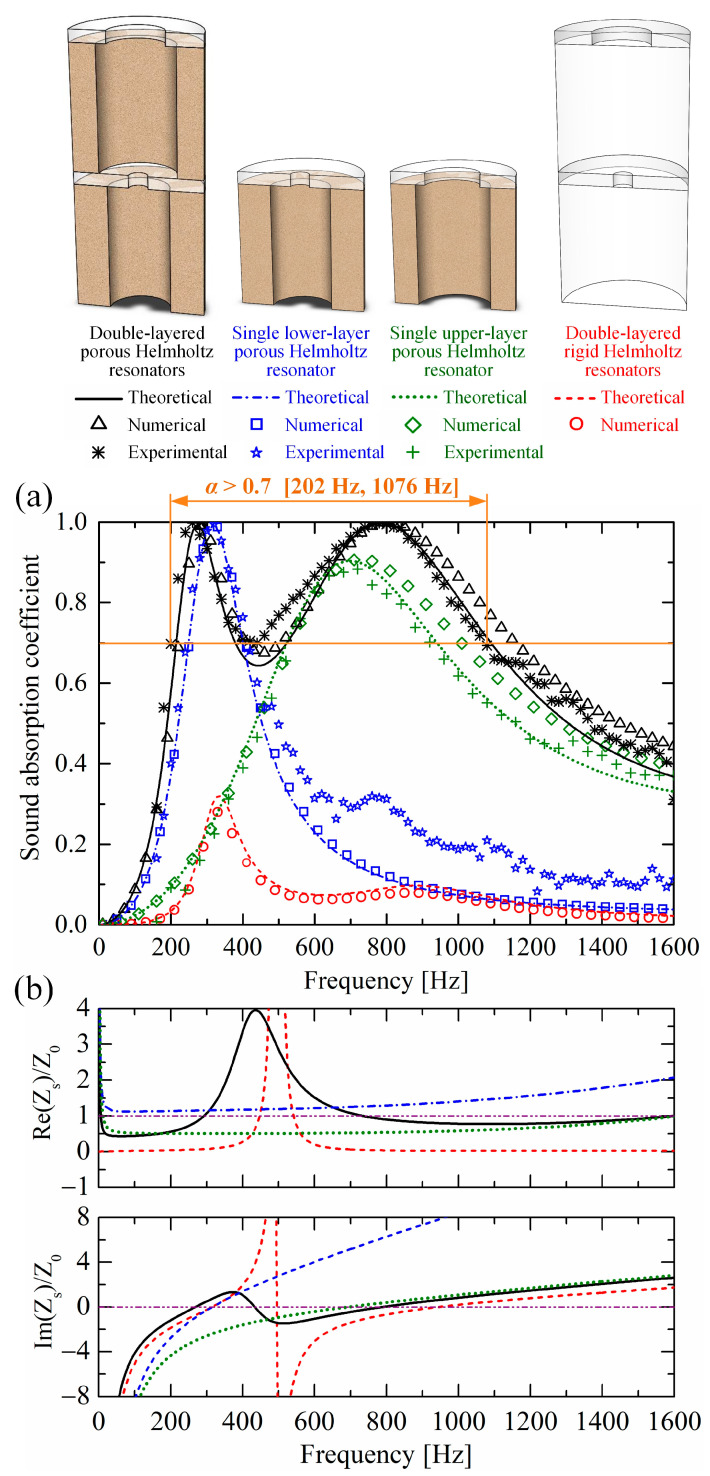
(**a**) Sound absorption performances and (**b**) normalized surface impedances of the double-layered porous Helmholtz resonators, single lower-layer porous Helmholtz resonator, single upper-layer porous Helmholtz resonator, and double-layered rigid Helmholtz resonators.

Figure 4 compares the sound absorption performances and surface impedances (normalized with air’s characteristic impedance) of the designed double-layered porous Helmholtz resonators, the corresponding rigid Helmholtz resonators, the single lower-layer porous Helmholtz resonator, and the upper-layer porous Helmholtz resonator. The sound absorption of the perforated porous materials and the porous material matrix are shown in Appendix B. The real part of the surface impedance represents the acoustic resistance, denoting the sound energy dissipating capacity; the imaginary part represents the acoustic reactance, denoting the sound energy storage capacity. Good sound absorption requires absorbers to allow more incident sound energy to enter the absorber and then be dissipated, which corresponds to a good balance between the resistance and reactance. Seen from Equation (14), the best balance is achieved when the imaginary part and the real part of the normalized surface impedance simultaneously reach 0 and 1, respectively, which corresponds to the perfect sound absorption (i.e., an absorption coefficient of 1).

As shown in Figure 4a, good agreements are achieved among the theoretical, numerical, and experimental results for all the four structures. The double-layered porous structure has two absorption peaks at 262 Hz and 774 Hz from experimental results (at 284 Hz and 801 Hz from numerical results, and at 274 Hz and 785 Hz from theoretical results) with absorption coefficients over 0.99. At 262 Hz, the structure thickness is around the 1/11.8 of the sound wave wavelength in air, realizing subwavelength sound absorption. Meanwhile, the experimental absorption coefficient exceeds 0.7 from 202 Hz to 1076 Hz (corresponding to 874 Hz bandwidth), realizing a broadband sound absorption.

Comparing the double-layered porous Helmholtz resonators and the corresponding rigid Helmholtz resonators, the absorption ability is substantially enhanced after the addition of perforated porous materials. This is because the two orifice diameters of the rigid absorber are as large as $d_{t\_1}=7.5$ mm and $d_{t\_2}=22$ mm, leading to very weak acoustic resistance to dissipate incident sound energy. As shown in Figure 4b, when the red reactance line reaches the horizontal line valued 0, the red resistance line is much lower than the horizontal line valued 1.

Comparing the double-layered porous Helmholtz resonators and the single lower-layer porous Helmholtz resonator, the double-layered absorber has a lower-frequency absorption peak, which corresponds to its black reactance line reaching the horizontal line valued 0 earlier. This implies that the added upper-layer resonator with both larger perforation hole diameters ($d_{h\_2}$ and $d_{t\_2}$) functions as an “acoustic buffer layer”, which forms a graded structure, allowing more sound to enter the absorber and thus improving reactance.

Comparing the double-layered porous Helmholtz resonators and the single upper-layer porous Helmholtz resonator, the double-layered absorber has a higher absorption coefficient at the second peak around 800 Hz where the two lines reach 0. Seen from Figure 4b, their reactance lines are very close around 800 Hz; however, the resistance line of the double-layered absorber is much closer to 1. The resistance of the single upper-layer absorber is insufficient, implying that the addition of the back lower-layer porous Helmholtz resonator can supply required resistance, that is, enhance the sound energy dissipation ability.

**Figure 5:**
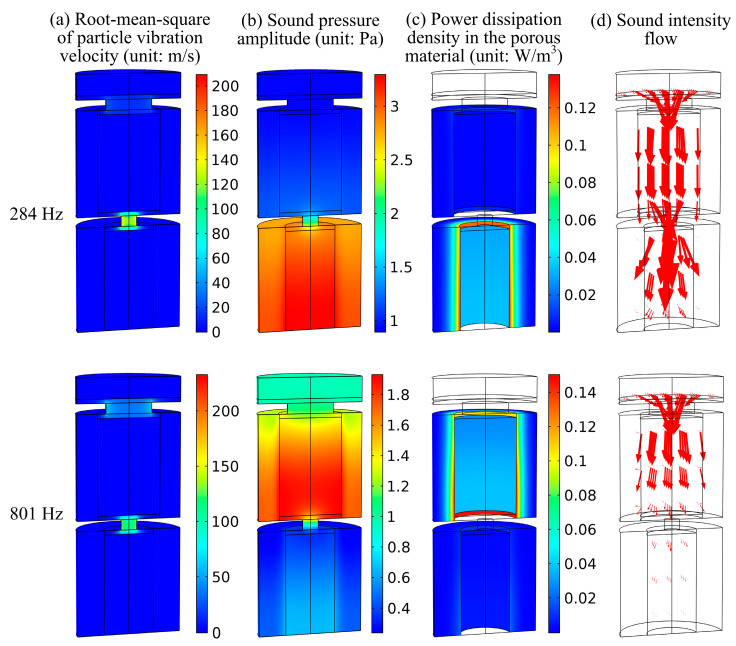
Color nephograms of (**a**) particle vibration velocity, (**b**) sound pressure amplitude, (**c**) power dissipation density $D_{p}$, and (**d**) sound intensity flow. The length and direction of the arrows in (**d**) represent the magnitude and direction of sound intensity. At 284 Hz and 801 Hz, the arrow sizes refer to the same reference scale.

To further clarify the absorption mechanisms, Figure 5 shows the nephograms of the sound intensity flow, root-mean-square (RMS) of the particle vibration velocity, sound pressure amplitude, and power dissipation density in the porous material at the two absorption peak frequencies of 284 Hz and 801 Hz from simulations. At 284 Hz, which is close to the resonance frequency of the lower-layer porous resonator, as shown in Figure 4a, the vibration velocity in the orifice and sound pressure in the cavity of the lower-layer are much larger than those in the upper-layer. The enhanced sound pressure promotes sound diffusion from the perforation hole into the neighboring resistive porous material domain, leading to substantial dissipation as shown in Figure 5c. As shown in Figure 5d, little sound intensity enters the porous material domain in the upper layer. On the contrary, at 801 Hz, which is close to the resonance frequency of the upper-layer porous resonator, Figure 5a shows that the vibration velocity in the orifice of the upper layer is enhanced in comparison with that at 284 Hz. And, as shown in Figure 5b, the sound pressure in the upper cavity is much larger than that in the lower cavity. The dominant energy dissipation area transfers to the upper perforated porous material, as shown in Figure 5c. Most of the incident sound energy does not enter the lower cavity, as shown in Figure 5d.

**Figure 6:**
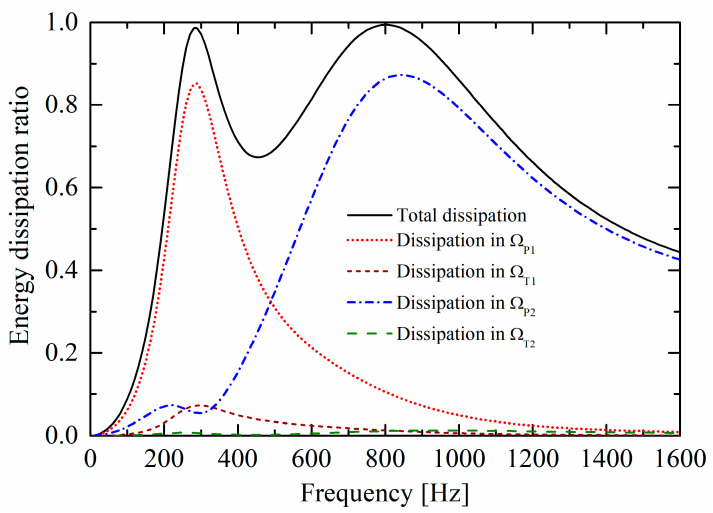
Ratios ($\gamma_{P1}$, $\gamma_{T1}$, $\gamma_{P2}$, and $\gamma_{T2}$ of the energy dissipations in the four domains $\Omega_{P1}$ (the porous material of the lower layer), $\Omega_{T1}$ (around the orifice of the lower layer), $\Omega_{P2}$ (the porous material of the upper layer), and $\Omega_{T2}$ (around the orifice of the upper layer) contributing to the total absorption of the incident sound energy.

Figure 6 compares the ratios of the energy dissipations in the four domains (Poroacoustics domains $\Omega_{P1}$ and $\Omega_{P2}$, Thermoviscous acoustics domains $\Omega_{T1}$ and $\Omega_{T2}$) contributing to the total absorption of the incident sound energy. Around 284 Hz, the porous material domain $\Omega_{P1}$ of the lower layer contributes the most dissipation, implying that Helmholtz resonance occurs. This corresponds to the dissipation nephogram in Figure 5c and sound intensity flow in Figure 5d. Similarly, around 801 Hz, the dominant energy dissipation area transfers to the porous material domain $\Omega_{P2}$ of the upper layer.

Different from conventional multi-layered micro-perforated panel absorbers dissipating sound energy by the orifices of sub-millimeter scale, Figure 6 demonstrates that this multi-layered porous Helmholtz resonators absorber with orifices of centimeter scale and millimeter scale dissipates sound energy mainly by inner perforated porous material. The larger orifice is easier to fabricate and less susceptible to blockage in harsh environments.

## 6. Parameter Study

Influences of orifice diameters and thicknesses of perforated rigid panels, thicknesses and perforation hole diameters of perforated porous materials, and porous material matrices on sound absorption performances are discussed in this section. The basic parameters are the same as those in the double-layer case described in Section 4 and Section 5. When discussing one parameter, all other parameters remain unchanged.

### 6.1. Influences of Orifice Diameters

**Figure 7:**
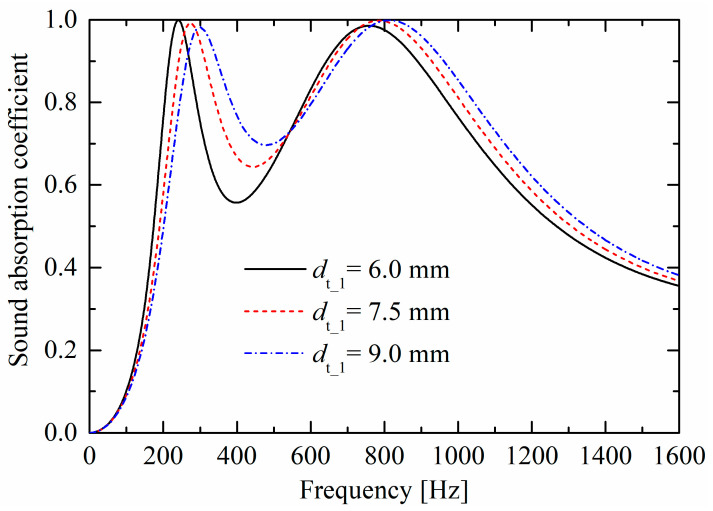
Influences of orifice diameter $d_{t\_1}$ of the lower layer on sound absorption performances.

**Figure 8:**
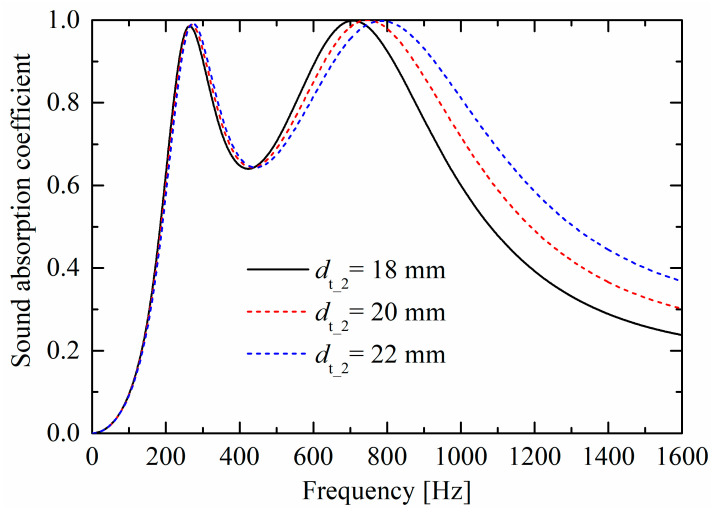
Influences of orifice diameter $d_{t\_2}$ of the upper layer on sound absorption performances.

Figure 7 and Figure 8 compare the influences of the orifice diameters ($d_{t\_1}$ and $d_{t\_2}$) of the lower layer and upper layer on sound absorption performances, respectively. As shown in Figure 7, as the orifice diameter decreases, the corresponding first absorption peak shifts towards lower frequencies, which is consistent with the second absorption peak shown in Figure 8. This trend is similar to that of a conventional rigid Helmholtz resonator with resonance frequency $c_{0}\sqrt{S_{\mathrm{neck}}/\left(V_{c}T_{\mathrm{neck}}\right)}/\left(2\pi \right)$, where $S_{neck}$ and $T_{\mathrm{neck}}$ are the cross-sectional area and thickness of the neck, respectively, and $V_{c}$ is the volume of the back cavity. In Figure 7, as the orifice diameter decreases, the second peak also slightly shifts towards lower frequencies, and the absorption curve valley decreases. However, as shown in Figure 8, variations in the upper-layer orifice diameter $d_{t\_2}$ have little influence on the absorption curve valley and the first absorption peak which corresponds to the lower layer.

### 6.2. Influences of Rigid Panel Thicknesses

**Figure 9:**
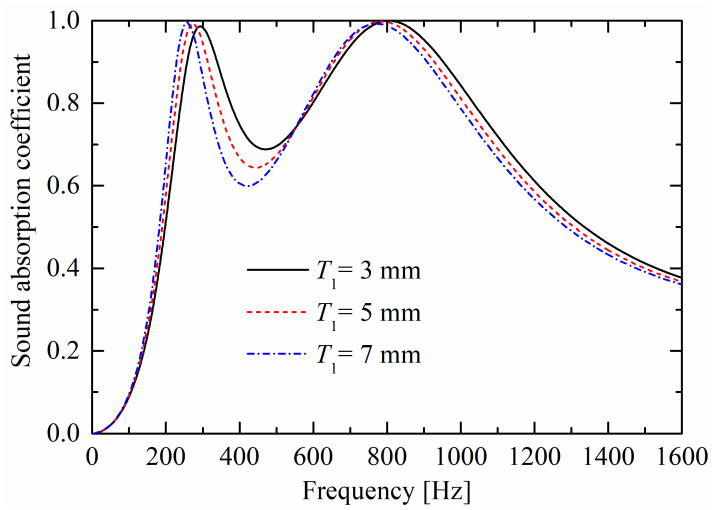
Influences of panel thickness $T_{1}$ of the lower layer on sound absorption performances.

**Figure 10:**
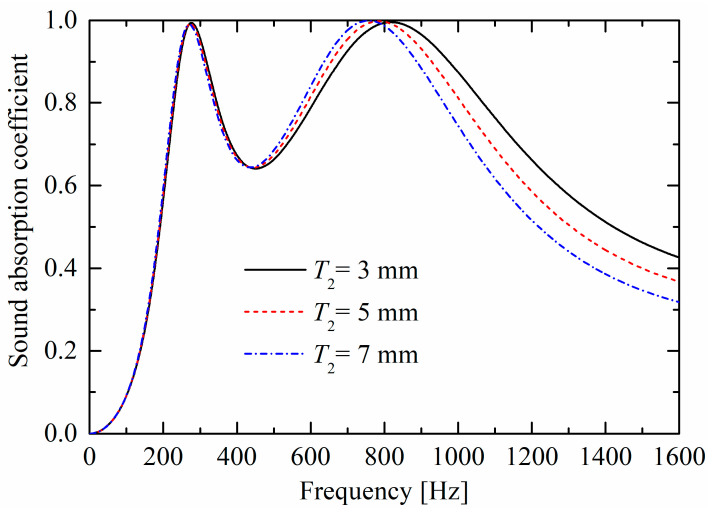
Influences of panel thickness $T_{2}$ of the upper layer on sound absorption performances.

As shown in Figure 9 and Figure 10, as rigid panel thicknesses ($T_{1}$ and $T_{2}$) increase, corresponding absorption peaks both move towards lower frequencies, owing to the increase of “air mass” in the neck (and constant “air spring” of the back cavity). The largest difference between the influences in Figure 9 and Figure 10 is that, as the thickness increases, the absorption curve valley in Figure 9 decreases; however, the valley is nearly unchanged in Figure 10, which is similar to the trend in Figure 8.

### 6.3. Influences of Perforated Porous Material Panel Thicknesses

**Figure 11:**
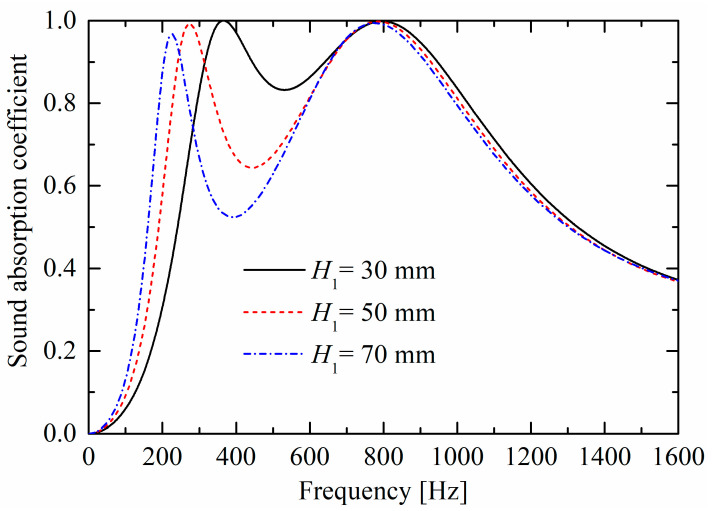
Influences of perforated porous material thickness $H_{1}$ of the lower layer on sound absorption performances.

**Figure 12:**
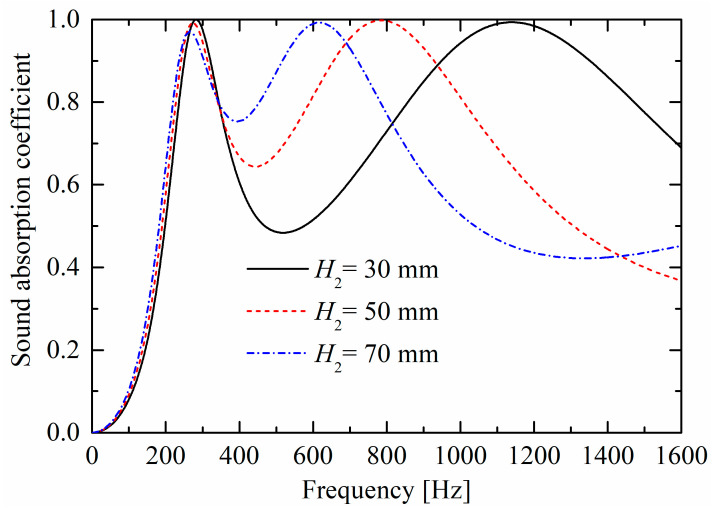
Influences of perforated porous material thickness $H_{2}$ of the upper layer on sound absorption performances.

As shown in Figure 11 and Figure 12, as perforated porous material panel thicknesses increase, the corresponding peaks move towards lower frequencies, owing to the decrease in the stiffness of the back cavity (i.e., the “air spring”). When the two absorption peaks become closer, the absorption valley increases.

### 6.4. Influences of Perforation Hole Diameters of Perforated Porous Materials

**Figure 13:**
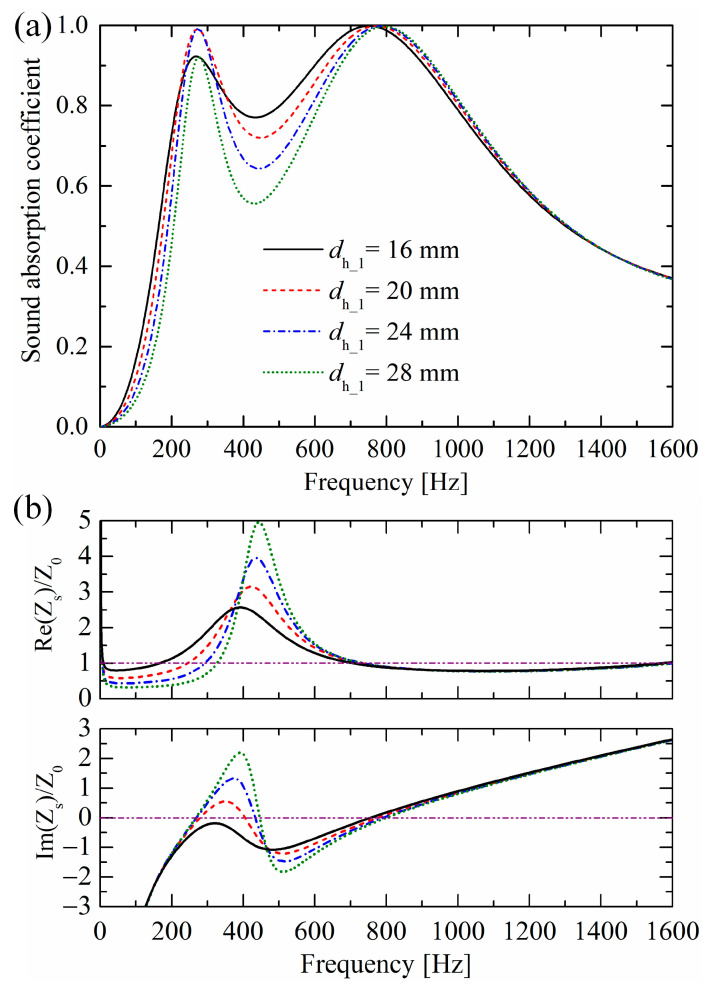
Influences of perforation hole diameter $d_{h\_1}$ of the lower-layer perforated porous material on (**a**) sound absorption performances and (**b**) normalized surface impedances.

As shown in Figure 13, as the perforation hole diameter $d_{h\_1}$ of the lower-layer perforated porous material decreases, the resistance around the corresponding first absorption peak frequency increases, and the first absorption peak first increases and then decreases, which implies that too large or too small resistance will break the impedance match with air. Moreover, as $d_{h\_1}$ decreases, the absorption valley increases.

**Figure 14:**
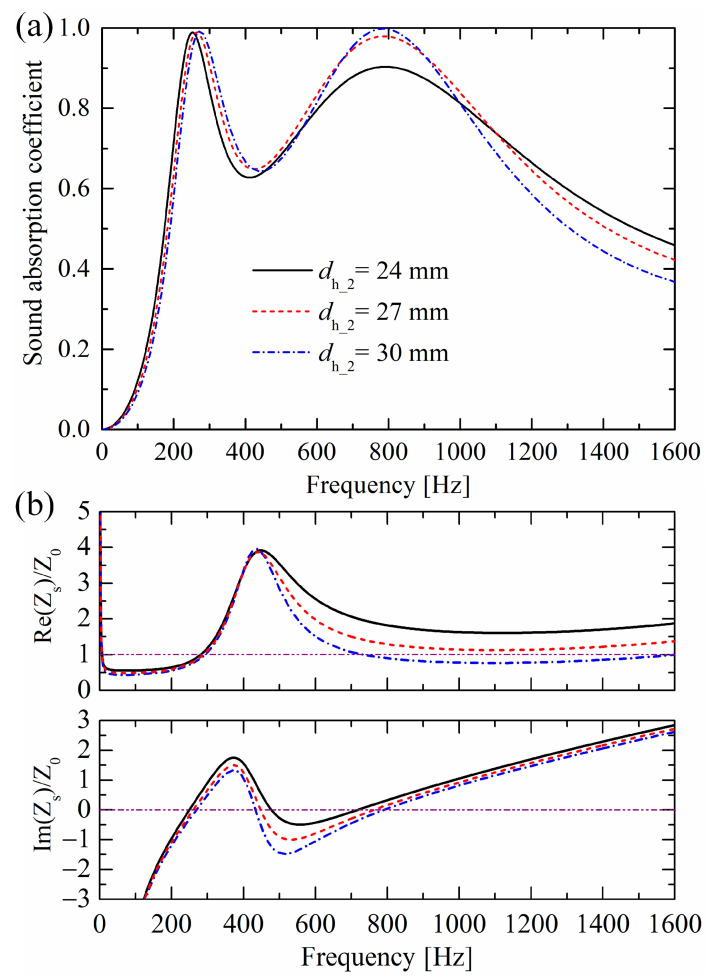
Influences of perforation hole diameter $d_{h\_2}$ of the upper-layer perforated porous material on (**a**) sound absorption performances and (**b**) normalized surface impedances.

Similarly, the perforation hole diameter $d_{h\_2}$ of the lower-layer perforated porous material mainly influences the second absorption peak, as shown in Figure 14. As $d_{h\_2}$ decreases, resistance increases. Moreover, $d_{h\_2}$ has a very slight influence on the absorption valley, which differs from the influences of $d_{h\_1}$.

### 6.5. Influences of Porous Material Matrices

**Figure 15:**
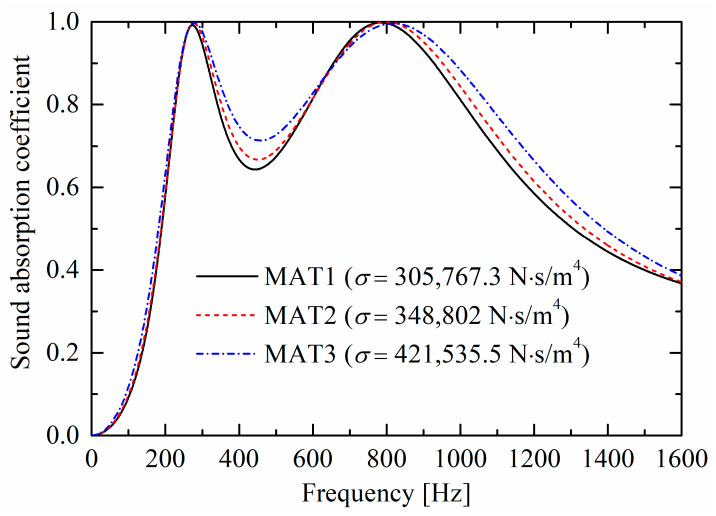
Influences of porous material matrices on sound absorption performances.

The static airflow resistivity σ is considered the dominant parameter in the JCA model for a porous material matrix of high porosity. Three kinds of ceramic fibrous material matrices with five JCA model parameters are listed in Table 1, marked as MAT1, MAT2, and MAT3, with the static airflow resistivity ranging from 305,767.3 N·s/m^4^ to 421,535.5 N·s/m^4^. As shown in Figure 15, as the flow resistivity increases, the absorption curve valley increases, and the two absorption peaks shift slightly towards higher frequencies.

## 7. Triple-Layered Porous Helmholtz Resonators

**Figure 16:**
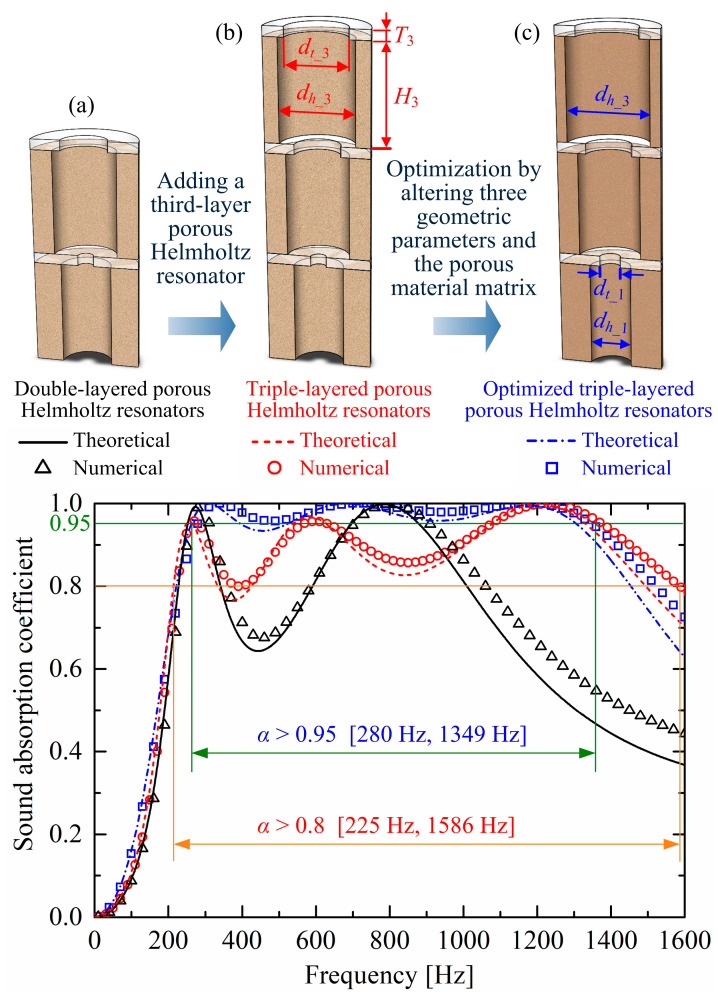
Sound absorption performance comparisons: (**a**) the double-layered porous Helmholtz resonators with MAT1 as the porous material matrix; (**b**) the triple-layered porous Helmholtz resonators obtained by adding a third-layer upon the absorber in (**a**), with $d_{t\_3}=30$ mm, $d_{h\_3}=36$ mm, $T_{3}=5$ mm, and $H_{3}=50$ mm; (**c**) an optimized triple-layer absorber by altering three geometric parameters and the porous material matrix of the absorber in (**b**), with $d_{h\_3}=40$ mm, $d_{t\_1}=10$ mm, $d_{h\_1}=20$ mm, and MAT3 as the porous material matrix.

A triple-layered porous Helmholtz resonators absorber shown in Figure 16b is obtained by adding a third-layer resonator to the double-layered porous Helmholtz resonators absorber depicted in Figure 16a with the same parameters as those in Figure 4. The third-layer resonator has the geometric parameters $d_{t\_3}=30$ mm, $d_{h\_3}=36$ mm, $T_{3}=5$ mm, and $H_{3}=50$ mm. The porous material matrix in the third-layer resonator is still MAT1 in Table 1. As shown in the absorption performances in Figure 16, this addition generates a third absorption peak, and the second absorption peak moves towards a lower frequency, contributing to a broader band: numerical absorption coefficient curve is over 0.8 from 225 Hz to 1586 Hz.

Moreover, an optimized triple-layer absorber with higher absorption coefficients in Figure 16c is obtained by altering three geometric parameters and the porous material matrix of the absorber, as shown in Figure 16b. The three new parameters are $d_{h\_3}=40$ mm, $d_{t\_1}=10$ mm, and $d_{h\_1}=20$ mm, instead of the original $d_{h\_3}=36$ mm, $d_{t\_1}=7.5$ mm, and $d_{h\_1}=24$ mm, respectively. The porous material matrix is MAT3, as listed in Table 1, instead of the original MAT1. After optimization, the absorption performance is improved with coefficients over 0.95 from 280 Hz to 1349 Hz. It should be noted that this optimization was not achieved through automatic parameter tuning by computer programs, but rather through manual parameter adjustments based on the influence patterns of parameters on sound absorption performances. As shown in Figure 7 and Figure 13, Figure 14 and Figure 15, appropriately increasing the orifice diameter $d_{t\_1}$ of the lower perforated rigid panel, the perforation diameter $d_{h\_2}$ of the upper perforated porous material, and the airflow resistivity σ of the porous material matrix, while reducing the perforation diameter $d_{h\_1}$ of the lower perforated porous material, will elevate the valley of the sound absorption coefficient curve.

## 8. Concluding Remarks

The sound absorption performance and mechanisms of multi-layered porous Helmholtz resonators are studied in this paper. The theoretical model is developed mainly based on the double porosity theory, perforated panel theory, and impedance translation theorem, and then is validated by the finite element simulations and experimental measurements of a double-layer test sample. The sample has two nearly perfect absorption peaks, realizing sub-wavelength sound absorption and broadband sound absorption.

The absorption mechanisms are first analyzed through the impedance perspective: the upper-layer with larger orifices acts as an “acoustic buffer layer”, which forms a graded structure, letting more sound enter the absorber and thus improving reactance for the first absorption peak; the addition of the back lower-layer porous Helmholtz resonator can supply required resistance, enhancing the sound energy dissipation ability at the second absorption peak.

Absorption mechanisms are further analyzed through nephograms of the sound pressure, particle vibration velocity, power dissipation density, and sound intensity flows using finite element simulations. At each absorption peak frequency, the sound pressure and velocity increase intensely in the corresponding Helmholtz resonator, promoting the entry of sound energy into resistive porous materials and leading to large dissipation.

Parameter studies show that this absorber maintains high absorption peaks across a wide range of orifice diameters, perforated rigid panel thicknesses, and perforated porous material panel thicknesses. In particular, the parameter variations in the upper-layer perforated rigid panel have little influence on the absorption curve valley. An appropriately increase in the airflow resistivity of the porous material matrix can increase the sound absorption curve valley.

Finally, an optimized triple-layer porous Helmholtz resonators absorber is presented, showing an ultra-broadband over an absorption coefficient of 0.95 from 280 Hz to 1349 Hz, with a total thickness of merely 16.5 mm. Compared with conventional multi-layer micro-perforated panels with orifices of sub-millimeter scale, the proposed multi-layered porous Helmholtz resonators have orifices of millimeter scale and centimeter scale, which are easier to fabricate and less susceptible to blockage in harsh environments, offering an alternative solution for low-frequency and broadband sound absorption.

## Figures and Tables

**Table 1:** Acoustic parameters of porous material matrices.

	$\phi_{m}$	σ [N·s/m^4^]	$\alpha_{\infty }$	Λ [μm]	$\Lambda^{\prime }$ [μm]
MAT1	0.89	305,767.3	3.7	21.6	53.6
MAT2	0.875	348,802	3.54	14.67	41.54
MAT3	0.89	421,535.5	2.835	11.65	38.38

**Figure A1:**
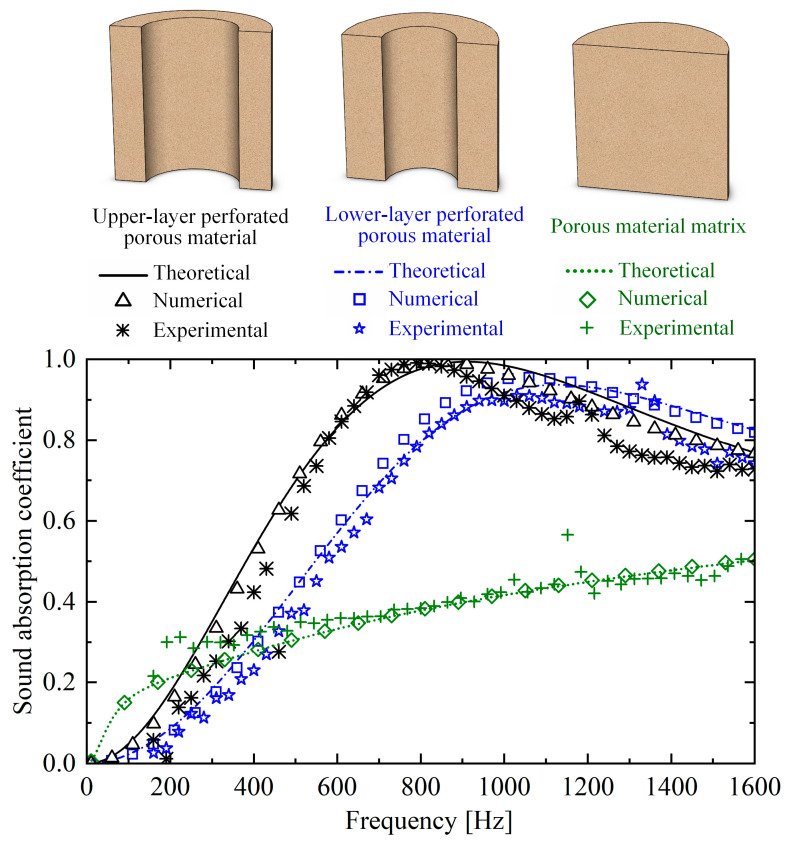
The theoretical, numerical, and experimental sound absorption coefficients of the upper-layer and lower-layer perforated porous materials in Figure 3 and the porous material matrix with same thickness of 50 mm.

## Data Availability

The original contributions presented in this study are included in the article. Further inquiries can be directed to the corresponding authors.

## Appendix A. Governing Equations and Sound Power Dissipation Densities in the “Thermoviscous Acoustics Domain” of the Finite Element Model

The built-in governing equations of the “Thermoviscous acoustics domain” considering the viscous and thermal dissipations in the software COMSOL Multiphysics 5.3 are set as follows:

(A1) $$\begin{array}{l} j\omega \rho_{v}+\nabla \cdot \left(\rho_{0}u\right)=0\\ j\omega \rho_{0}u=\nabla \cdot \left\{-pI+\eta \left[\nabla u+{\left(\nabla u\right)}^{T}\right]-\left(\frac{2}{3}\eta -\eta_{B}\right)\left(\nabla \cdot u\right)I\right\}\\ \rho_{0}C_{p}\left(j\omega T_{v}+u\cdot \nabla T_{0}\right)-\alpha_{p}T_{0}\left(j\omega p+u\cdot \nabla P_{0}\right)=\nabla \cdot \left(\kappa \nabla T_{v}\right) \end{array}$$

where $\rho_{v}$ is the air density variation, $T_{v}$ is the temperature variation, $T_{0}=293.15$ K is the equilibrium temperature, u is the particle vibration velocity field, I is the identity matrix, ${\left(\cdot \right)}^{T}$ denotes transposition, and $\eta_{B}$ is the bulk viscosity of air and is set 0 Pa·s here. $\rho_{v}$ is related to the air equilibrium density $\rho_{0}$:

(A2) $$\rho_{v}=\rho_{0}\left(\beta_{T}p-\alpha_{p}T_{v}\right)$$

where $\alpha_{p}$ (unit: 1/K) is the isobaric thermal expansion coefficient, and $\beta_{T}$ (unit: 1/Pa) is the isothermal compressibility, defined as

(A3) $$\alpha_{p}=-\frac{1}{\rho_{0}}\cdot \frac{\partial \rho_{0}\left(T_{0},P_{0}\right)}{\partial T_{0}}$$

(A4) $$\beta_{T}=-\frac{1}{\rho_{0}}\cdot \frac{\partial \rho_{0}\left(T_{0},P_{0}\right)}{\partial P_{0}}$$

The viscous power dissipation density and thermal power dissipation density $D_{T\_v}$ and $D_{T\_t}$ in the software are written as

(A5) $$D_{T\_v}=\frac{\chi :\nabla u^{*}}{2}$$

(A6) $$D_{T\_t}=\frac{\kappa \left[\nabla T_{v}\cdot \nabla T_{v}^{*}\right]}{2T_{0}}$$

where χ denotes the stress tensor with its components written as

(A7) $$\chi_{mn}=\eta \left(\frac{\partial u_{m}}{\partial x_{n}}+\frac{\partial u_{n}}{\partial x_{m}}\right)-\left(\frac{2}{3}\eta -\eta_{B}\right)\left(\frac{\partial u_{k}}{\partial x_{k}}\right)\delta_{mn}$$

following the Einstein summation convention, where $\delta_{mn}$ denotes the Kronecker delta function.

## Appendix B. Sound Absorption Performances of the Perforated Porous Materials and Porous Material Matrix

Figure A1 shows good agreements are achieved among the theoretical, numerical, and experimental sound absorption coefficients of the upper-layer and lower-layer perforated porous materials in Figure 3 and the porous material matrix with same thickness of 50 mm. After perforating holes in the porous material matrix, sound absorption performances are significantly improved.
